# Evaluation of Darolutamide (ODM201) Efficiency on Androgen Receptor Mutants Reported to Date in Prostate Cancer Patients

**DOI:** 10.3390/cancers13122939

**Published:** 2021-06-11

**Authors:** Nada Lallous, Oliver Snow, Christophe Sanchez, Ana Karla Parra Nuñez, Bei Sun, Ahmed Hussain, Joseph Lee, Helene Morin, Eric Leblanc, Martin E. Gleave, Artem Cherkasov

**Affiliations:** Vancouver Prostate Centre, Department of Urologic Sciences, University of British Columbia, 2660 Oak St., Vancouver, BC V6H 3Z6, Canada; nlallous@prostatecentre.com (N.L.); oliver_snow@sfu.ca (O.S.); christophe.sanchez997@gmail.com (C.S.); anaparranunez@gmail.com (A.K.P.N.); bei.sun@outlook.com (B.S.); ahmedbhus@gmail.com (A.H.); jlee@prostatecentre.com (J.L.); hmorin@prostatecentre.com (H.M.); eleblanc@prostatecentre.com (E.L.); m.gleave@ubc.ca (M.E.G.)

**Keywords:** androgen receptor, antagonists, castration-resistant prostate cancer (CRPC), darolutamide, drug resistance, mutations

## Abstract

**Simple Summary:**

Prostate cancer (PCa) is the most commonly diagnosed non-skin cancer in men and one of the leading causes of cancer-related death. The driver of PCa proliferation and growth is the androgen receptor (AR) and inhibiting this receptor is the standard of care for patients, following surgery or radiotherapy. Unfortunately, the effectiveness of current therapeutics is temporary, with the cancer eventually developing drug resistance. Among the mechanisms of resistance are the arising mutations in the AR that make the receptor promiscuously activated by drugs or non-specific ligands, thus promoting cancer progression. The aim of this study is to characterize the responses of 44 AR mutants, derived from PCa patients, to available steroids that activate the receptor as well as to various treatments currently used in the clinic. This work will help create a tool to guide the medical team in selecting the best personalized treatment option for each patient.

**Abstract:**

Resistance to drug treatments is common in prostate cancer (PCa), and the gain-of-function mutations in human androgen receptor (AR) represent one of the most dominant drivers of progression to resistance to AR pathway inhibitors (ARPI). Previously, we evaluated the in vitro response of 24 AR mutations, identified in men with castration-resistant PCa, to five AR antagonists. In the current work, we evaluated 44 additional PCa-associated AR mutants, reported in the literature, and thus expanded the study of the effect of darolutamide to a total of 68 AR mutants. Unlike other AR antagonists, we demonstrate that darolutamide exhibits consistent efficiency against all characterized gain-of-function mutations in a full-length AR. Additionally, the response of the AR mutants to clinically used bicalutamide and enzalutamide, as well as to major endogenous steroids (DHT, estradiol, progesterone and hydrocortisone), was also investigated. As genomic profiling of PCa patients becomes increasingly feasible, the developed “AR functional encyclopedia” could provide decision-makers with a tool to guide the treatment choice for PCa patients based on their AR mutation status.

## 1. Introduction

The emergence of drug resistance in prostate cancer (PCa) is a prominent factor of its progression to castration-resistant PCa (CRPC). Notably, human androgen receptor (AR) remains the main driver in both PCa and CRPC [1]. Therefore, the use of androgen deprivation therapy (ADT) has been the standard of care for PCa patients, relying on direct inhibition of AR signalling axes [2]. The remarkable plasticity [3] of the AR in response to targeted therapies is now well recognized, and there is an impressive repertoire of AR-genomic and non-genomic mechanisms of treatment escape, including gain-of-function mutations in the androgen-binding site (ABS) of the receptor [4,5,6,7,8]. It used to be widely accepted that such resistance occurs as the result of treatment selection of pre-existing drug-resistant sub-clones of AR. However, a recent study [9] demonstrated that downregulation of DNA mismatch repair (MMR) and homologous recombination (HR) play a significant role in adaptive mutability in colorectal cancers, occurring as a response to therapeutic pressure. Such adaptive mutability has also been reported for epidermal growth factor receptor (EGFR) in non-small cell lung cancers [10]. Similarly, in PCa there is a 4-fold difference in mutation rates in metastases compared to primary tumours [11], and a subset of metastatic PCa presents a hypermutated MMR leading to oncogene activation and tumour heterogeneity [12]. Therefore, adaptive mutability could rapidly contribute to the emergence of acquired drug-resistant sub-clones in advanced PCa.

Our group and others have previously demonstrated that the treatment pressure directed at the AR by clinically used AR antagonists leads to drug-induced mutations in the AR androgen-binding site (ABS), changing the pocket characteristics and inducing receptor activation by adrenal/prostate androgens, by steroidal and non-steroidal ligands and, notably, by the AR antagonists themselves [13,14,15,16]. Recently, Ledt et al. analyzed the circulating tumour cell free DNA (cfDNA) of 892 patients with advanced PCa, and demonstrated that 32% of patients with AR alterations present nonsynonymous mutations (SNVs or indels) [17]. Similarly, by analyzing the *cBio* cancer genomics portal data base [18,19], we found that the frequency of AR mutants can vary between patient cohorts and can reach up to 15% in metastatic CRPC [4,20]. We also reported the results of functional characterization of 24 AR mutants identified in liquid biopsies from CRPC patients or reported in the literature, and demonstrated that all these mutants exhibited resistance to at least one of four available AR antagonists, including hydroxyflutamide, bicalutamide, enzalutamide and apalutamide [13].

The remarkable plasticity of the AR under selective pressure of AR pathway inhibition (ARPI), coupled with the marked heterogeneity and negative prognostic significance of its cfDNA mutants, indicates that there is no “one size fits all” treatment for PCa patients. Furthermore, the results of our initial functional characterization of clinically observed AR mutants clearly indicate the need for novel AR antagonist(s) capable of inhibiting all forms of AR mutants.

**Figure 1:**
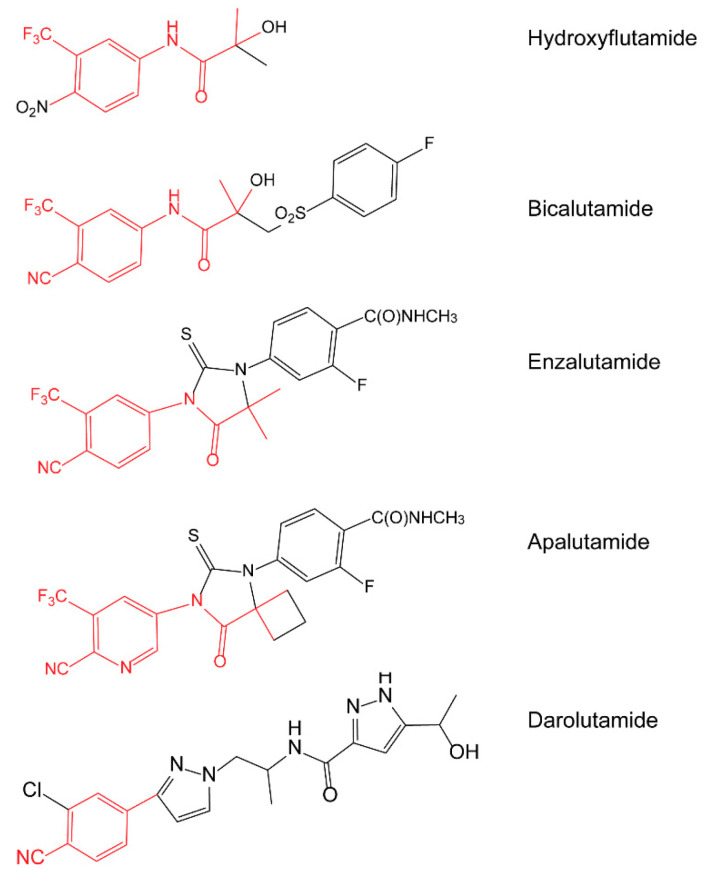
Chemical structures of clinically used AR antagonists.

Darolutamide, a structurally distinct AR antagonist compared to ABS antagonists hydroxyflutamide, bicalutamide, enzalutamide and apalutamide (Figure 1), showed complete inhibition of several documented AR-resistant mutants [21] and might provide broader antagonist activity with emergent AR mutants. Hence, we evaluated the inhibition of 44 PCa-associated AR mutants identified in the literature and public databases by darolutamide. Additionally, the response of the AR mutants to most clinically used bicalutamide and enzalutamide, as well as to major endogenous steroids (DHT, estradiol, progesterone and hydrocortisone), was investigated.

## 2. Materials and Methods

### 2.1. Constructs

Full-length human AR (WT-AR) was encoded on a pcDNA3.1 expression plasmid (Life Technologies, Carlsbad, CA, USA). The AR point mutations were generated using the QuikChange II Site-Directed Mutagenesis Kit (Agilent Technologies, Santa Clara, CA, USA) as per manufacturer’s instructions using WT-AR as the template. The mutagenic oligonucleotide primers were designed individually with the desired mutation in the middle of the primer with ~10–15 bases of correct sequence on both sides (the sequences of the used primers are presented in Table S1).

### 2.2. Steroid Activation Assay

PC3 cells lacking the AR and authenticated by Genetica using STR profiling were maintained in RPMI 1640 media (Life Technologies) and 5% FBS (Hyclone Thermo Fisher Scientific, Waltham, MA, USA) at 37 °C and 5% CO_2_. Cultures were routinely monitored for mycoplasma contamination. For the steroid activation assay, cells were seeded in 96-well plates (5000 cells/well) in RPMI 1640 medium with 5% charcoal-stripped serum (CSS) (Hyclone). After 24 h, cells were co-transfected with 25 ng of wild-type or mutated AR and 25 ng of the reporter plasmid pARR3-tk-luciferase using TransIT20/20 transfection reagent (3 μL/μg of DNA) (Mirus Bio LLC, Madison, WI, USA) in Opti-MEM serum-free media (Life Technologies) for 48 h according to manufacturer’s suggested protocol. Cells were stimulated with increasing concentrations of DHT, estradiol, progesterone or hydrocortisone in 100% ethanol (0 to 500 nM). Control cells were treated with 100% ethanol alone. At 24 h after treatment, the medium was aspirated off and the cells were lysed by adding 60 μL of 1× passive lysis buffer (Promega, Madison, WI, USA) followed by shaking at room temperature for 15 min and two freeze/thaw cycles at −80 °C. Twenty microlitres of lysate from each well was transferred onto a 96-well white flat-bottom plate (Corning, NY, USA) and the luminescence signal was measured after adding 50 μL of luciferase assay reagent (Promega). The chemical oxidation of luciferin into oxyluciferin by the luciferase is accompanied by light production that can be quantified as luminescence by a TECAN M200Pro instrument. Each concentration was assayed in quadruplicate, *n* = 4, with at least 3 biological replicates. For each steroid, results were averaged and normalized by expressing them as a percentage of WT AR activity.

### 2.3. AR Inhibition Assay

PC3 cells were seeded and transfected as described above. At 48 h after transfection, medium was aspirated and replaced with medium containing 0.1 nM R1881 (PerkinElmer, Waltham, MA, USA) and either 0.1% DMSO (control) or serial dilutions of increasing concentrations of AR inhibitors ranging from 0 to 25 μM. After 24 h, cells were lysed and AR-dependent luciferase activity was quantified. Each concentration was assayed in quadruplicate, *n* = 4, with at least 3 biological replicates. Results were averaged and normalized by expressing them as a percentage of WT AR activity. Darolutamide was purchased from MedKoo Biosciences (Cat#206514; Morrisville, NC, USA).

### 2.4. Western Blotting

Twenty microlitres of each of 8 replicates of DMSO/control-treated lysate from the luciferase assay were pooled and the total amount of protein was assayed by bicinchoninic acid assay (BCA) (Pierce™, Appleton, WI, USA). Equal amounts of protein samples were loaded on a 10% SDS-PAGE gel and electrophoresed at 120 V for 90 min. Proteins were transferred to PVDF membrane (Millipore, Burlington, MA, USA) at 25 V for 15 min using a TransBlot^®^ Turbo™ Transfer System (Bio-Rad Laboratories, Hercules, CA, USA). The membrane was then blocked for 30 min at room temperature with 5% non-fat skim milk in TBS, followed by incubation with 1/1000 dilution of AR (441) mouse monoclonal antibody (sc-7305, Santa Cruz Biotechnologies, Dallas, TX, USA) and GAPDH Antibody (G-9) HRP mouse monoclonal antibody (sc-365062, Santa Cruz Biotechnologies) overnight at 4 °C. Membranes were then washed and incubated with 1/1000 dilution of Donkey Anti-Mouse IgG Polyclonal Antibody (IRDye^®^ 680RD; 925-68072; LI-COR Biosciences, Lincoln, NE, USA) for 1 h at room temperature, washed 3 times with TBS 0.1% Tween 20 (Sigma-Aldrich, St. Louis, MO, USA) and bands visualized using Odyssey Li-Cor Scanner.

## 3. Results

Characterization of resistance-associated AR mutants is critically important for predicting and monitoring patients’ response to therapy and ultimately, for the development of evidence-based precision oncology practices [22,23]. In this work, we have expanded the list of functionally characterized AR mutants with an additional 44 variants reported in “The Androgen Receptor Gene Mutations Database World Wide Web Server” of McGill University [24]. These were associated with PCa and mainly localized in either the ligand-binding domain (LBD) or the DNA-binding domain (DBD) of the receptor (Table S1). We evaluated the response of these 44 AR mutants to increasing concentrations of four steroids (dihydrotestosterone (DHT), progesterone, hydrocortisone and estradiol) as well as three clinically used AR antagonists, including the first-generation bicalutamide, the second-generation enzalutamide and the most recently approved darolutamide [25].

### 3.1. AR Transcriptional Activation by Steroids

**Figure 2:**
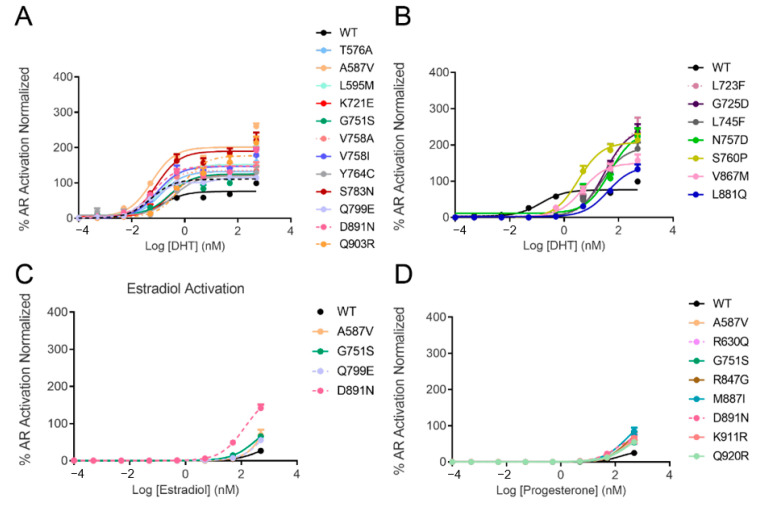
Steroid activation of AR mutants in comparison with the wild-type receptor in luciferase reporter assay. AR mutants that showed similar (**A**) or lower (**B**) affinities to DHT than WT but reached higher activation levels. Mutants that were better activated by estradiol and progesterone, compared to wild-type, are shown in (**C**,**D**), respectively. None of the mutants were activated by hydrocortisone. The graphs represent the average ± SE of three independent experiments with four replicates each. The activity of each mutant in the presence of a steroid was normalized to the wild type stimulated by 500 nM of DHT.

The response of AR mutants to increasing concentrations of DHT was measured using a luciferase reporter transcription assay in PC3 cells transiently transfected with either wild-type (WT) or mutated AR. The expression level of all of the mutants was evaluated by Western blotting (Figure S1). Some mutants were activated by lower concentrations of DHT than WT (EC50 = 0.14 nM), such as A587V (EC50 = 0.06 nM), K631T (EC50 = 0.05 nM), Q671R (EC50 = 0.05 nM), V756A/I (EC50 = 0.07 and 0.09 nM, respectively), S783N (EC50 = 0.09 nM), Q799E (EC50 = 0.05 nM) and D891N (EC50 = 0.07 nM) (Table 1 and Table S2). Twelve mutants (T576A, A587V, L595M, K721E, G751S, V758A/I, Y764C, S783N, Q799E, D891N and Q903R) with similar or higher affinities than WT were over-stimulated and reached higher levels of transcriptional activation up to 2 times more than WT (Figure 2A and Table S2). Another set of mutants (L723F, G725D, L745F, N757D, S760P, V867M and L881Q) were stimulated by higher concentrations of DHT with EC50s ranging from three to ~40 nM; however, they reached higher levels of activity. One such example is L723F (EC50 = 43 nM), showing 2.5-fold increased activity compared to the wild-type AR (Figure 2B and Table S2). These new results illustrate the very heterogeneous response of AR mutants to DHT, ranging from complete insensitivity to hyper-activation.

We further evaluated the response of 44 studied AR variants to activation by estradiol, progesterone and hydrocortisone. The wild-type AR was only mildly stimulated by progesterone with EC50 in the range of 150 nM and was not activated with estradiol or hydrocortisone at concentrations as high as 500 nM (Table 1). Notably, D891N was the only mutant in the cohort that demonstrated detectable transcriptional activity in the presence of 100 nM estradiol, and at 500 nM it peaked with 5-fold increased transactivation (compared to the wild type). Three other mutants—A587V, G751S and Q799E—demonstrated modestly enhanced activation by estradiol (Figure 2C and Table S2).

Of the 44 studied AR mutants, eight exhibited slightly higher levels of activation by progesterone compared to WT-AR (A587V, R630Q, G751S, R847G, M887I, D891N, K911R and Q920R) (Figure 2D and Table S2). Neither WT-AR nor any of the tested AR variants demonstrated any transcriptional activity when cells were stimulated by 500 nM hydrocortisone. Three mutants in particular—A587V, G751S and D891N—made the receptor promiscuous to activation by progesterone and estradiol while providing a better affinity or higher activity in the presence of the DHT.

### 3.2. AR Transcriptional Inhibition by AR Antagonists

**Figure 3:**
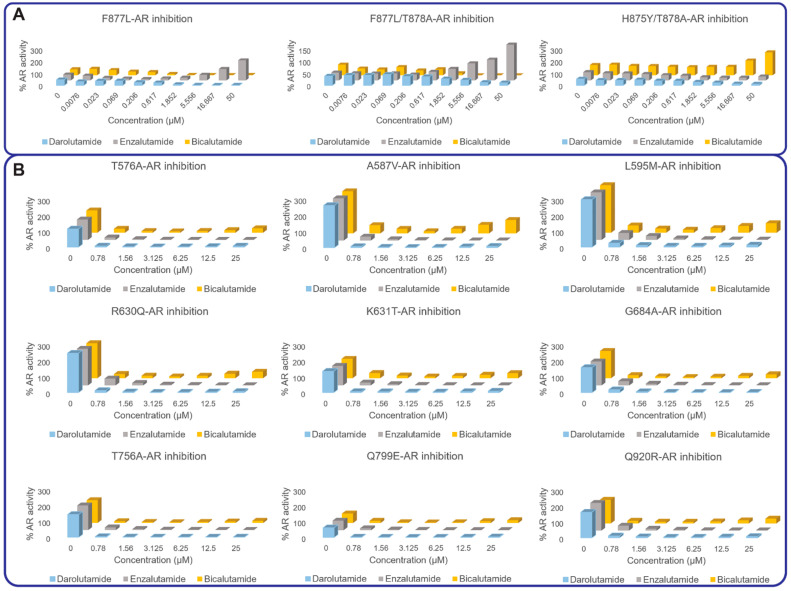
AR mutants showing partial or full agonist response in presence of high concentrations of AR antagonists. (**A**) Previously characterized AR mutants activated by enzalutamide or bicalutamide treatment. (**B**) Newly characterized AR mutants showing signs of activation in presence of bicalutamide. Each concentration was assayed in quadruplicate, *n* = 4, with a biological replicate of *n* = 3. Results were averaged and normalized by expressing them as a percentage of WT-AR activity.

We previously characterized CRPC-associated AR mutants that were mainly located in the vicinity of the receptor’s ligand-binding site [13,21]. All the previously studied AR mutants demonstrated activation by at least one clinically used AR antagonist. In this study, we evaluated the response of an additional 44 PCa-associated AR mutants to darolutamide along with the drugs broadly used in the clinic, bicalutamide and enzalutamide (Figure 3 and Table S2). None of the mutants exhibited full activation with the tested drugs, with some of them demonstrating a partially agonistic response toward bicalutamide at concentrations above 6 µM. Of those, A587V and L595M presented the most prominent partial agonist behavior corresponding to AR reactivation at concentrations as low as 3.25 µM of bicalutamide. All mutants were efficiently inhibited by the second-generation AR antagonists enzalutamide and darolutamide, with no significant reactivation at concentrations up to 25 µM (Figure 3 and Table S2).

## 4. Discussion

Acquired drug resistance represents a paramount danger for PCa patients. There are different mechanisms underlying the development of such resistance [3], yet AR remains one of the most dominant drivers in most scenarios. Thus, AR gain-of-function mutations have been extensively reported in various clinical cohorts (Table S1) [4,13,17,18]. Such mutations can promiscuously activate the receptor by non-specific steroids and/or by antiandrogen drugs. Thus, it has been previously observed that 24 patient-derived CRPC-associated AR mutants can be activated by at least one of the clinically used AR antagonists, including hydroxyflutamide, bicalutamide, enzalutamide and apalutamide. Only darolutamide later demonstrated complete suppression of all 24 AR mutants with signs of reactivation only in presence of V716M [21] (Figure 3). In this work, we expand the study with 44 mutants located in both the LBD and DBD domains of the AR and described in the McGill database [24], bringing the total number of mutants to 68.

Ten of the studied mutants—C620Y, L723F, G725D, L745F, M750I, N757D, S760P, S792P, S866P and V867M—did not demonstrate any detectible transcriptional activity in the presence of 0.1 nM of the synthetic active anabolic androgenic steroid, the standard AR agonist R1881, in our transcriptional inhibition assay. Out of those ten mutants, C620Y, M750I and S866P did not show any transcriptional activation also in the presence of 500 nM DHT, while the other mutants (L723F, G725D, L745F, N757D, S760P, S792P and V867M) required higher concentrations of DHT to reach and surpass the wild-type level of stimulation (Table 1, Table S2 and Figure 2). Only four mutants showed some activation in presence of estrogen and eight were stimulated by progesterone to similar or higher levels as wild-type AR (Figure 2). Two mutants in particular, A587V and D891N, made the receptor promiscuously activated by the DHT, estradiol and progesterone (Figure 2).

In recent studies where patients were exposed to CYP17A1 inhibitor abiraterone and/or to first- and second-generation antiandrogens, the recurrent mutations were L702H, W742C/L, H875Y, F877L and T878A/S, that gained a significant spotlight and were characterized in our previous study [13]. Herein we report on documented but less noted AR mutants, yet most of them still demonstrated various degrees of activation by bicalutamide (Figure 3 and Table S2). Darolutamide and enzalutamide demonstrated generally very potent inhibition of all the studied mutants.

**Figure 4:**
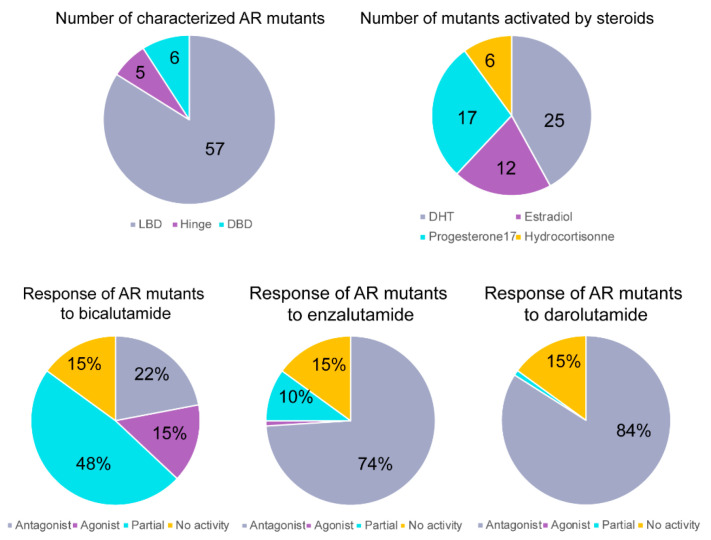
Summary of functional characterization of AR mutants. We functionally characterized 68 AR mutants, mainly in the LBD, and few in the DBD and Hinge regions. Twenty-five of the studied mutants were activated by DHT, 12 by estradiol, 17 by progesterone and 6 by hydrocortisone to higher levels than wild-type AR. AR mutants showed different response profiles in presence of first- (bicalutamide) and second (enzalutamide)-generation AR antagonists and the newly approved darolutamide. Of the characterized mutants, 15% did not show any activity in our assay.

In summary, out of 68 experimentally evaluated AR mutants (24 reported in the previous works and 44 presented in the current study), 25 demonstrated enhanced activation by DHT, 17 by progesterone, 12 by estradiol and 6 by hydrocortisone, compared to the wild-type receptor (Figure 4). The first-generation bicalutamide behaved as a partial or complete agonist for the majority, 43 out of 68, of studied AR mutants (63% of mutants). The second-generation antiandrogen, enzalutamide, demonstrated full or partial activation of eight mutant variants, while the structurally distinct and most recently approved darolutamide demonstrated significant activation in only one mutant at concentrations up to 25 µM [21], which identifies a sequencing opportunity for this drug in men with progressive CRPC with a gain-of-function mutation in the AR under selective pressure of first-line ARPIs. Evaluating those mutants in mouse models is certainly needed to confirm the in vitro data reported here and may be a direction for our future work.

## 5. Conclusions

Emergent AR mutations in men with advanced PCa treated with ARPI promote CRPC progression. The incidence of AR mutations was estimated to be around 15% for CRPC patients [4] and the availability of circulating tumour DNA assays now provide a sensitive method to serially detect (and treat) the emergence of resistant AR mutants. This current work expanded the list of experimentally evaluated AR mutants with 44 additional examples (bringing the total to 68) and quantified their response to four major endogenous steroids and three clinically used AR antagonists, including darolutomide. Among these, only darolutamide demonstrated complete inhibition of 67 out of the 68 studied AR mutant variants, with no significant signs of partial of full activation at even higher concentrations.

## Figures and Tables

**Table 1:** The activation of AR mutants by various steroids. The EC50 values of the activation by DHT, estradiol, progesterone and hydrocortisone are reported for the wild-type AR and the 44 studied mutants. For steroid activation, we tested a concentration range up to 500 nM; therefore, mutants showing no activation or very weak activation in the studied range are presented with EC50 values > 500 nM.

AR Construct	EC_50_ of DHT (nM)	EC_50_ of Estradiol (nM)	EC_50_ of Progesterone (nM)	EC_50_ of Hydrocortisone (nM)
WT	0.14	>500	144	>500
T576A	0.12	>500	115	>500
K581R	weak	>500	>500	>500
A587V	0.06	>500	168	>500
A588S	1.15	>500	>500	>500
L595M	0.14	>500	167	>500
C620Y	>500	>500	>500	>500
R630Q	0.10	>500	151	>500
K631T	0.05	>500	118	>500
E666D	0.11	>500	269	>500
Q671R	0.05	>500	170	>500
G684A	0.17	>500	148	>500
K721E	0.27	>500	>500	>500
A722T	0.67	>500	>500	>500
L723F	42.66	>500	>500	>500
G725D	31.12	>500	>500	>500
L745F	23.27	>500	>500	>500
A749T	17.56	>500	>500	>500
A749V	140.3	>500	>500	>500
M750I	>500	>500	>500	>500
G751S	0.11	>500	134	>500
F755L	0.66	>500	>500	>500
T756A	0.43	>500	>500	>500
N757D	2.67	>500	>500	>500
V758A	0.07	>500	>500	>500
V758I	0.09	>500	>500	>500
S760P	3.33	>500	>500	>500
Y764C	0.40	>500	>500	>500
S783N	0.09	>500	162	>500
S792P	>500	>500	>500	>500
Q799E	0.05	>500	147	>500
I800T	0.55	>500	>500	>500
R847G	0.17	>500	267	>500
S866P	>500	>500	>500	>500
V867M	4.62	>500	>500	>500
E873Q	weak	>500	>500	>500
D880G	0.85	>500	>500	>500
L881Q	40.58	>500	>500	>500
M887I	0.17	>500	253	>500
D891N	0.07	134.2	135	>500
A897T	0.15	>500	154	>500
Q903R	0.74	>500	>500	>500
G910E	0.25	>500	>500	>500
K911R	0.11	>500	295	>500
Q920R	0.15	>500	320	>500

## Data Availability

Not applicable.

## Supplementary Materials

The following are available online at https://www.mdpi.com/article/10.3390/cancers13122939/s1, Table S1: AR mutations identified in the ligand-binding domain (LBD) or the DNA-binding domain (DBD), Table S2: The response of PCa-associated mutants to increasing concentrations of steroids (dihydrotestosterone, progesterone, estradiol and hydrocortisone) and AR antagonists (bicalutamide, enzalutamide and darolutamide), Figure S1: Western blot showing expression level of the PCa-associated AR mutants.

## References

[1] Feng Q., He B. (2019). Androgen Receptor Signaling in the Development of Castration-Resistant Prostate Cancer. Front. Oncol..

[2] Shore N.D. (2020). Current and Future Management of Locally Advanced and Metastatic Prostate Cancer. Rev. Urol..

[3] Snow O., Lallous N., Singh K., Lack N., Rennie P., Cherkasov A. (2019). Androgen receptor plasticity and its implications for prostate cancer therapy. Cancer Treat. Rev..

[4] Robinson D., Van Allen E.M., Wu Y.-M., Schultz N., Lonigro R.J., Mosquera J.-M., Montgomery B., Taplin M.-E., Pritchard C.C., Attard G. (2015). Integrative Clinical Genomics of Advanced Prostate Cancer. Cell.

[5] Antonarakis E.S., Lu C., Wang H., Luber B., Nakazawa M., Roeser J.C., Chen Y., Mohammad T.A., Chen Y., Fedor H.L. (2014). AR-V7 and Resistance to Enzalutamide and Abiraterone in Prostate Cancer. N. Engl. J. Med..

[6] Montgomery R.B., Mostaghel E.A., Vessella R., Hess D.L., Kalhorn T.F., Higano C.S., True L.D., Nelson P.S. (2008). Maintenance of Intratumoral Androgens in Metastatic Prostate Cancer: A Mechanism for Castration-Resistant Tumor Growth. Cancer Res..

[7] Sailer V., Eng K.W., Zhang T., Bareja R., Pisapia D.J., Sigaras A., Bhinder B., Romanel A., Wilkes D., Sticca E. (2019). Integrative Molecular Analysis of Patients With Advanced and Metastatic Cancer. JCO Precis. Oncol..

[8] Watson P.A., Arora V.K., Sawyers C.L. (2015). Emerging mechanisms of resistance to androgen receptor inhibitors in prostate cancer. Nat. Rev. Cancer.

[9] Russo M., Crisafulli G., Sogari A., Reilly N.M., Arena S., Lamba S., Bartolini A., Amodio V., Magrì A., Novara L. (2019). Adaptive mutability of colorectal cancers in response to targeted therapies. Science.

[10] Hata A.N., Niederst M.J., Archibald H.L., Gomez-Caraballo M., Siddiqui F.M., Mulvey H.E., Maruvka Y.E., Ji F., Bhang H.-E.C., Radhakrishna V.K. (2016). Tumor cells can follow distinct evolutionary paths to become resistant to epidermal growth factor receptor inhibition. Nat. Med..

[11] Tandefelt D.G., de Bono J. (2020). Circulating cell-free DNA: Translating prostate cancer genomics into clinical care. Mol. Asp. Med..

[12] Ritch E., Fu S.Y., Herberts C., Wang G., Warner E.W., Schönlau E., Taavitsainen S., Murtha A.J., Vandekerkhove G., Beja K. (2019). Identification of Hypermutation and Defective Mismatch Repair in ctDNA from Metastatic Prostate Cancer. Clin. Cancer Res..

[13] Lallous N., Volik S.V., Awrey S., Leblanc E., Tse R., Murillo J., Singh K., Azad A.A., Wyatt A.W., LeBihan S. (2016). Functional analysis of androgen receptor mutations that confer anti-androgen resistance identified in circulating cell-free DNA from prostate cancer patients. Genome Biol..

[14] Bohl C.E., Gao W., Miller D.D., Bell C.E., Dalton J.T. (2005). Structural basis for antagonism and resistance of bicalutamide in prostate cancer. Proc. Natl. Acad. Sci. USA.

[15] Bohl C.E., Miller D.D., Chen J., Bell C.E., Dalton J.T. (2005). Structural Basis for Accommodation of Nonsteroidal Ligands in the Androgen Receptor. J. Biol. Chem..

[16] Korpal M., Korn J.M., Gao X., Rakiec D.P., Ruddy D.A., Doshi S., Yuan J., Kovats S.G., Kim S., Cooke V.G. (2013). An F876L Mutation in Androgen Receptor Confers Genetic and Phenotypic Resistance to MDV3100 (Enzalutamide). Cancer Discov..

[17] Ledet E.M., Lilly M.B., Sonpavde G., Lin E., Nussenzveig R.H., Barata P.C., Yandell M., Nagy R.J., Kiedrowski L., Agarwal N. (2019). Comprehensive Analysis of AR Alterations in Circulating Tumor DNA from Patients with Advanced Prostate Cancer. Oncologist.

[18] Cerami E., Gao J., Dogrusoz U., Gross B.E., Sumer S.O., Aksoy B.A., Jacobsen A., Byrne C.J., Heuer M.L., Larsson E. (2012). The cBio Cancer Genomics Portal: An Open Platform for Exploring Multidimensional Cancer Genomics Data. Cancer Discov..

[19] Gao J., Aksoy B.A., Dogrusoz U., Dresdner G., Gross B., Sumer S.O., Sun Y., Jacobsen A., Sinha R., Larsson E. (2013). Integrative Analysis of Complex Cancer Genomics and Clinical Profiles Using the cBioPortal. Sci. Signal..

[20] Abida W., Cyrta J., Heller G., Prandi D., Armenia J., Coleman I., Cieslik M., Benelli M., Robinson D., Van Allen E.M. (2019). Genomic correlates of clinical outcome in advanced prostate cancer. Proc. Natl. Acad. Sci. USA.

[21] Borgmann H., Lallous N., Ozistanbullu D., Beraldi E., Paul N., Dalal K., Fazli L., Haferkamp A., Lejeune P., Cherkasov A. (2018). Moving Towards Precision Urologic Oncology: Targeting Enzalutamide-resistant Prostate Cancer and Mutated Forms of the Androgen Receptor Using the Novel Inhibitor Darolutamide (ODM-201). Eur. Urol..

[22] Azad A.A., Volik S.V., Wyatt A.W., Haegert A., Le Bihan S., Bell R.H., Anderson S.A., McConeghy B., Shukin R., Bazov J. (2015). Androgen Receptor Gene Aberrations in Circulating Cell-Free DNA: Biomarkers of Therapeutic Resistance in Castration-Resistant Prostate Cancer. Clin. Cancer Res..

[23] Ku S.-Y., Gleave M., Beltran H. (2019). Towards precision oncology in advanced prostate cancer. Nat. Rev. Urol..

[24] Gottlieb B., Beitel L.K., Nadarajah A., Paliouras M., Trifiro M. (2012). The androgen receptor gene mutations database: 2012 update. Hum. Mutat..

[25] Fizazi K., Shore N., Tammela T.L., Ulys A., Vjaters E., Polyakov S., Jievaltas M., Luz M., Alekseev B., Kuss I. (2019). Darolutamide in Nonmetastatic, Castration-Resistant Prostate Cancer. N. Engl. J. Med..

