# Supplementary material for: Evaluation of Darolutamide (ODM201) Efficiency on Androgen Receptor Mutants Reported to Date in Prostate Cancer Patients
Source: Cancers (Basel). 2021 Jun 11;13(12):2939. doi: 10.3390/cancers13122939 (PMC8230763; doi:10.3390/cancers13122939)
Supplement: Supplementary file 1 [file cancers-13-02939-s001.zip › cancers-1251001 Table S1.pdf]

Table S1. AR mutations identified in the ligand binding domain (LBD) or the DNA binding domain (DBD).

AR mutant	AR region	Identified in	Reference	Primers 5'-3'
T576A	DBD	PCa patient relapsing after leuprorelin and flutamide treatment and withbone metastasis	1, 2	FW: GTCACTATGGAGCTCTCGCATGTGGAAGCTGCAAG<br>RV: CTTGCAGCTTCCACATGCGAGAGCTCCATAGTGAC
K581R	DBD	Metastatic specimens of PCa patients	2	FW: CTCACATGTGGAAGCTGCAGGGTCTTCTTCAAAAGAG<br>RV: CTCTTTTGAAGAAGACCCTGCAGCTTCCACATGTGAG
A587V	DBD	Metastatic specimens of PCa patients	2	FW: GGTCTTCTTCAAAAGAGTCGCTGAAGGGAAACAGA<br>RV: TCTGTTTCCCTTCAGCGACTCTTTTGAAGAAGACC
A588S	DBD	Metastatic specimens of PCa patients	2	FW: GGTCTTCTTCAAAAGAGCCAGTGAAGGGAAACAGAAGTAC<br>RV: GTACTTCTGTTTCCCTTCACTGGCTCTTTTGAAGAAGACC
L595M	DBD	Liver metastases of patient treated with bicalutamide	3	FW: AAGGGAAACAGAAGTACATGTGCGCCAGCAGAAA<br>RV: TTTCTGCTGGCGCACATGTACTTCTGTTTCCCTT
C620Y	DBD	PCa patient with lymph node metastasis	2, 4	FW: TCTTGTCGTCTTCGGAAATATTATGAAGCAGGGATGAC<br>RV: GTCATCCCTGCTTCATAATATTTCCGAAGACGACAAGA
R630Q	Hinge	PCa patient with lymph node metastasis	5, 6	FW: GATGACTCTGGGAGCCCAGAAGCTGAAGAAACTTG<br>RV: CAAGTTTCTTCAGCTTCTGGGCTCCCAGAGTCATC
K631T	Hinge	PCa patient before ADT	5, 7	FW: ACTCTGGGAGCCCGGACGCTGAAGAAACTTG<br>RV: CAAGTTTCTTCAGCGTCCGGGCTCCCAGAGT
E666D	Hinge	Liver metastases of patient treated with bicalutamide	3	FW: AGAAGCTGACAGTGTCACACATTGATGGCTATGAATGTC<br>RV: GACATTCATAGCCATCAATGTGTGACACTGTCAGCTTCT
Q671R	LBD	Primary tumor from PCa patient before ADT	7	FW: ATTGAAGGCTATGAATGTCGGCCCATCTTTCTGAATGTC<br>RV: GACATTCAGAAAGATGGGCCGACATTCATAGCCTTCAAT
G684A	LBD	Patient with localized PCa	8	FW: AAGCCATTGAGCCAGCTGTAGTGTGTGCTGG<br>RV: CCAGCACACACTACAGCTGGCTCAATGGCTT
K721E	LBD	PCa patient with bone metastasis	9, 10	FW: GGTCAAGTGGGCCGAGGCCTTGCCTGG<br>RV: CCAGGCAAGGCCTCGGCCCACTTGACC
A722T	LBD	Patients with metastatic prostate cancer in relapse after orchiectomy or the administration of an LHRH agonist	11	FW: TCAAGTGGGCCAAGACCTTGCCTGGCTTC<br>RV: GAAGCCAGGCAAGGTCTTGGCCCACTTGA
L723F	LBD	Patient with localized PCa	12, 13	FW: AAGTGGGCCAAGGCCTTCCCTGGCTTCCGCA<br>RV: TGCGGAAGCCAGGGAAGGCCTTGGCCCACTT
G725D	LBD	Patients with metastatic prostate cancer in relapse after orchiectomy or the administration of an LHRH agonist	11	FW: CCAAGGCCTTGCCTGACTTCCGCAACTTACA<br>RV: TGTAAGTTGCGGAAGTCAGGCAAGGCCTTGG
L745F	LBD	Latent carcinomas from Japanese males	14	FW: GTACTCCTGGATGGGGTTCATGGTGTTTGCCAT<br>RV: ATGGCAAACACCATGAACCCCATCCAGGAGTAC

A749T	LBD	Metastatic specimens of PCa patients	2, 15	FW: GATGGGGCTCATGGTGTTTACCATGGGCTG<br>RV: CAGCCCATGGTAAACACCATGAGCCCCATC
A749V	LBD	Latent carcinomas from Japanese males	14	FW: ATGGGGCTCATGGTGTTTGTCATGGGCTGGCGAT<br>RV: ATCGCCAGCCCATGACAAACACCATGAGCCCCAT
M750I	LBD	Latent carcinomas from Japanese males and orchiectomy-Bicalutamide-Refractory Prostate Cancer	14, 16	FW: GCTCATGGTGTTTGCCATAGGCTGGCGATC<br>RV: GATCGCCAGCCTATGGCAAACACCATGAGC
G751S	LBD	Latent carcinomas from Japanese males	14	FW: CATGGTGTTTGCCATGAGCTGGCGATCCTTCAC<br>RV: GTGAAGGATCGCCAGCTCATGGCAAACACCATG
F755L	LBD	Latent carcinomas from Japanese males	14	FW: CCATGGGCTGGCGATCCTTAACCAATGTCAACTC<br>RV: GAGTTGACATTGGTTAAGGATCGCCAGCCCATGG
T756A	LBD	Latent carcinomas from Japanese males	14	FW: GGCTGGCGATCCTTCGCCAATGTCAACTCCA<br>RV: TGGAGTTGACATTGGCGAAGGATCGCCAGCC
N757D	LBD	PCa tumors from bone-marrow biopsies after ADT withdrawal	17	FW: CTGGCGATCCTTCACCGATGTCAACTCCAGGAT<br>RV: ATCCTGGAGTTGACATCGGTGAAGGATCGCCAG
V758A	LBD	Metastatic specimens of PCa patients	2	FW: CGATCCTTCACCAATGCCAACTCCAGGATGCTC<br>RV: GAGCATCCTGGAGTTGGCATTGGTGAAGGATCG
V758I	LBD	Patients with local tumor recurrence	18	FW: TGGCGATCCTTCACCAATATCAACTCCAGGATGC<br>RV: GCATCCTGGAGTTGATATTGGTGAAGGATCGCCA
S760P	LBD	Latent carcinomas from Japanese males	14	FW: CCTTCACCAATGTCAACCCCAGGATGCTCTACTTC<br>RV: GAAGTAGAGCATCCTGGGGTTGACATTGGTGAAGG
Y764C	LBD	Latent carcinomas from Japanese males	14	FW: CTCCAGGATGCTCTGCTTCGCCCCTGATC<br>RV: GATCAGGGGCGAAGCAGAGCATCCTGGAG
S783N	LBD	Primary tumor from PCa patient before ADT	7	FW: TGCACAAGTCCCGGATGTACAACCAGTGTGTCC<br>RV: GGACACACTGGTTGTACATCCGGGACTTGTGCA
S792P	LBD	Primary tumor from PCa patient before ADT	7	FW: CCGAATGAGGCACCTCCCTCAAGAGTTTGGATG<br>RV: CATCCAAACTCTTGAGGGAGGTGCCTCATTCGG
Q799E	LBD	Prostate carcinoma before treatment	19	FW: CAAGAGTTTGGATGGCTCGAGATCACCCCCCAGGAATTC<br>RV: GAATTCCTGGGGGGTGATCTCGAGCCATCCAAACTCTTG
I800T	LBD	Patient with localized PCa	12	FW: AGAGTTTGGATGGCTCCAAACCACCCCCCAGG<br>RV: CCTGGGGGGTGGTTTGGAGCCATCCAAACTCT
R847G	LBD	Metastatic specimens of PCa patients	2	FW: ATCGTATCATTGCATGCAAAGGAAAAAATCCCACATCCTGC<br>RV: GCAGGATGTGGGATTTTTTCCTTTGCATGCAATGATACGAT
S866P	LBD	Metastatic specimens of PCa patients	2	FW: CCAAGCTCCTGGACCCCGTGCAGCCTATT<br>RV: AATAGGCTGCACGGGGTCCAGGAGCTTGG
V867M	LBD	Patients with local tumor recurrence and latent carcinomas	14, 18	FW: AGCTCCTGGACTCCATGCAGCCTATTGCG<br>RV: CGCAATAGGCTGCATGGAGTCCAGGAGCT
E873Q	LBD	PCa patient after cyproterone acetate and chemotherapy	20	FW: CGTGCAGCCTATTGCGAGACAGCTGCATCA<br>RV: TGATGCAGCTGTCTCGCAATAGGCTGCACG

D880G	LBD	PCa tumors from bone-marrow biopsies after ADT withdrawal	17	FW: GCTGCATCAGTTCACTTTTGGCCTGCTAATCAAGTCAC<br>RV: GTGACTTGATTAGCAGGCCAAAAGTGAACTGATGCAGC
L881Q	LBD	Patients with metastatic prostate cancer in relapse after orchiectomy or the administration of an LHRH agonist	11	FW: CATCAGTTCACTTTTGACCAGCTAATCAAGTCACACATG<br>RV: CATGTGTGACTTGATTAGCTGGTCAAAAGTGAACTGATG
M887I	LBD	PCa patient after orchiectomy and bicalutamide	20	FW: ACCTGCTAATCAAGTCACACATAGTGAGCGTGGAC<br>RV: GTCCACGCTCACTATGTGTGACTTGATTAGCAGGT
D891N	LBD	PCa patient after LHRH agonist	21	FW: CACACATGGTGAGCGTGAACTTTCCGGAAATGATG<br>RV: CATCATTTCCGGAAAGTTCACGCTCACCATGTGTG
A897T	LBD	Patients with metastatic prostate cancer in relapse after orchiectomy or the administration of an LHRH agonist	11	FW: GGACTTTCCGGAAATGATGACGGAGATCATCTCTGTGCAAG<br>RV: CTTGCACAGAGATGATCTCCGTCATCATTTCCGGAAAGTCC
Q903R	LBD	Patients with metastatic prostate cancer in relapse after orchiectomy or the administration of an LHRH agonist	11	FW: GGCAGAGATCATCTCTGTGAGAGTGCCCAAGATCCTTTCT<br>RV: AGAAAGGATCTTGGGCACTCTCACAGAGATGATCTCTGCC
G910E	LBD	Latent carcinomas from Japanese males	14	FW: GCCCAAGATCCTTTCTGAGAAAGTCAAGCCCATCT<br>RV: AGATGGGCTTGACTTTCTCAGAAAGGATCTTGGGC
K911R	LBD	PCa patient after refractory to hormone therapy	22	FW: GCCCAAGATCCTTTCTGGGAGAGTCAAGCCCATC<br>RV: GATGGGCTTGACTCTCCCAGAAAGGATCTTGGGC
Q920R	LBD	Metastatic specimens of PCa patients	2	FW: ATCTATTTCCACACCCGGTGAAGGATCCACTAGT<br>RV: ACTAGTGGATCCTTCACCGGGTGTGGAAATAGAT

1. Monge A, Jagla M, Lapouge G, et al. Unfaithfulness and promiscuity of a mutant androgen receptor in a hormone-refractory prostate cancer. *Cellular and molecular life sciences : CMLS*. Feb 2006;63(4):487-97. doi:10.1007/s00018-005-5471-y
2. Marcelli M, Ittmann M, Mariani S, et al. Androgen receptor mutations in prostate cancer. *Cancer research*. Feb 15 2000;60(4):944-9.
3. Steinkamp MP, O'Mahony OA, Brogley M, et al. Treatment-dependent androgen receptor mutations in prostate cancer exploit multiple mechanisms to evade therapy. *Cancer research*. May 15 2009;69(10):4434-42. doi:10.1158/0008-5472.can-08-3605
4. Nazareth LV, Stenoien DL, Bingman WE, 3rd, et al. A C619Y mutation in the human androgen receptor causes inactivation and mislocalization of the receptor with concomitant sequestration of SRC-1 (steroid receptor coactivator 1). *Molecular endocrinology (Baltimore, Md)*. Dec 1999;13(12):2065-75. doi:10.1210/mend.13.12.0382
5. Haelens A, Tanner T, Denayer S, Callewaert L, Claessens F. The hinge region regulates DNA binding, nuclear translocation, and transactivation of the androgen receptor. *Cancer research*. May 1 2007;67(9):4514-23. doi:10.1158/0008-5472.can-06-1701
6. Wang C, Uchida T. [Androgen receptor gene mutations in prostate cancer]. *Nihon Hinyokika Gakkai zasshi The japanese journal of urology*. May 1997;88(5):550-6. doi:10.5980/jpnjurol1989.88.550

7. Tilley WD, Buchanan G, Hickey TE, Bentel JM. Mutations in the androgen receptor gene are associated with progression of human prostate cancer to androgen independence. *Clinical cancer research : an official journal of the American Association for Cancer Research*. Feb 1996;2(2):277-85.
8. Koivisto P, Kononen J, Palmberg C, et al. Androgen receptor gene amplification: a possible molecular mechanism for androgen deprivation therapy failure in prostate cancer. *Cancer research*. Jan 15 1997;57(2):314-9.
9. Duff J, McEwan IJ. Mutation of histidine 874 in the androgen receptor ligand-binding domain leads to promiscuous ligand activation and altered p160 coactivator interactions. *Molecular endocrinology (Baltimore, Md)*. Dec 2005;19(12):2943-54. doi:10.1210/me.2005-0231
10. Hay CW, McEwan IJ. The impact of point mutations in the human androgen receptor: classification of mutations on the basis of transcriptional activity. *PloS one*. 2012;7(3):e32514. doi:10.1371/journal.pone.0032514
11. Taplin ME, Bubley GJ, Shuster TD, et al. Mutation of the androgen-receptor gene in metastatic androgen-independent prostate cancer. *The New England journal of medicine*. May 25 1995;332(21):1393-8. doi:10.1056/nejm199505253322101
12. Sanchez D, Rosell D, Honorato B, Lopez J, Arocena J, Sanz G. Androgen receptor mutations are associated with Gleason score in localized prostate cancer. *BJU international*. Dec 2006;98(6):1320-5. doi:10.1111/j.1464-410X.2006.06438.x
13. Hiort O, Sinnecker GH, Holterhus PM, Nitsche EM, Kruse K. The clinical and molecular spectrum of androgen insensitivity syndromes. *American journal of medical genetics*. May 3 1996;63(1):218-22. doi:10.1002/(sici)1096-8628(19960503)63:1<218::aid-ajmg38>3.0.co;2-p
14. Takahashi H, Furusato M, Allsbrook WC, Jr., et al. Prevalence of androgen receptor gene mutations in latent prostatic carcinomas from Japanese men. *Cancer research*. Apr 15 1995;55(8):1621-4.
15. James AJ, Agoulnik IU, Harris JM, et al. A novel androgen receptor mutant, A748T, exhibits hormone concentration-dependent defects in nuclear accumulation and activity despite normal hormone-binding affinity. *Molecular endocrinology (Baltimore, Md)*. Dec 2002;16(12):2692-705. doi:10.1210/me.2001-0281
16. Haapala K, Hyytinen ER, Roiha M, et al. Androgen receptor alterations in prostate cancer relapsed during a combined androgen blockade by orchiectomy and bicalutamide. *Laboratory investigation; a journal of technical methods and pathology*. Dec 2001;81(12):1647-51. doi:10.1038/labinvest.3780378
17. Taplin ME, Rajeshkumar B, Halabi S, et al. Androgen receptor mutations in androgen-independent prostate cancer: Cancer and Leukemia Group B Study 9663. *Journal of clinical oncology : official journal of the American Society of Clinical Oncology*. Jul 15 2003;21(14):2673-8. doi:10.1200/jco.2003.11.102
18. Hyytinen ER, Haapala K, Thompson J, et al. Pattern of somatic androgen receptor gene mutations in patients with hormone-refractory prostate cancer. *Laboratory investigation; a journal of technical methods and pathology*. Nov 2002;82(11):1591-8. doi:10.1097/01.lab.0000038924.67707.75

19. Evans BA, Harper ME, Daniells CE, et al. Low incidence of androgen receptor gene mutations in human prostatic tumors using single strand conformation polymorphism analysis. *The Prostate*. Mar 1996;28(3):162-71. doi:10.1002/(sici)1097-0045(199603)28:3<162::aid-pros3>3.0.co;2-h
20. Chen G, Wang X, Zhang S, et al. Androgen receptor mutants detected in recurrent prostate cancer exhibit diverse functional characteristics. *The Prostate*. Jun 1 2005;63(4):395-406. doi:10.1002/pros.20191
21. Taplin ME, Bubley GJ, Ko YJ, et al. Selection for androgen receptor mutations in prostate cancers treated with androgen antagonist. *Cancer research*. Jun 1 1999;59(11):2511-5.
22. Watanabe M, Ushijima T, Shiraishi T, et al. Genetic alterations of androgen receptor gene in Japanese human prostate cancer. *Japanese journal of clinical oncology*. Dec 1997;27(6):389-93. doi:10.1093/jjco/27.6.389
