# Supplementary material for: Evaluation of Darolutamide (ODM201) Efficiency on Androgen Receptor Mutants Reported to Date in Prostate Cancer Patients
Source: Cancers (Basel). 2021 Jun 11;13(12):2939. doi: 10.3390/cancers13122939 (PMC8230763; doi:10.3390/cancers13122939)
Supplement: Supplementary file 1 [file cancers-13-02939-s001.zip › cancers-1251001 Table S2.pdf]

Table S2: The response of PCa-associated mutants to increasing concentrations of steroids (dihy-drotestosterone, progesterone, estradiol and hydrocortisone) and AR antagonists (bicalutamide, enzalutamide and darolutamide).

AR mutant	Activation				Inhibition		
	Dihydrotestosterone	Progesterone	Estradiol	Hydroxycortisone	Bicalutamide	Enzalutamide	Darolutamide
WT-AR	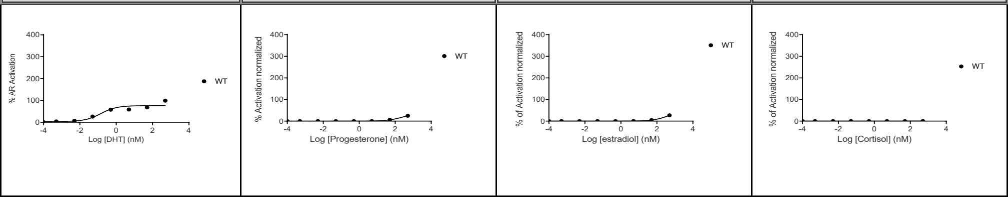				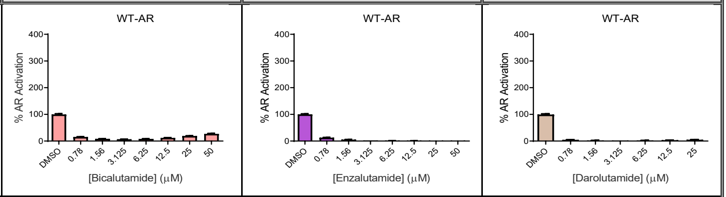		
T576A	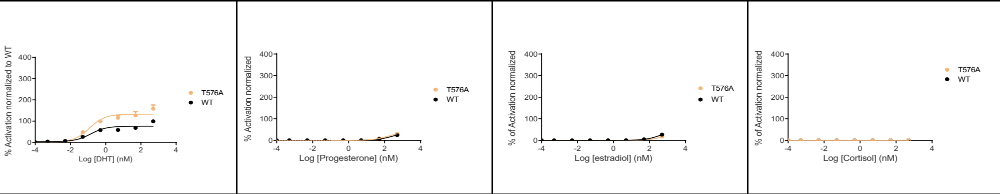				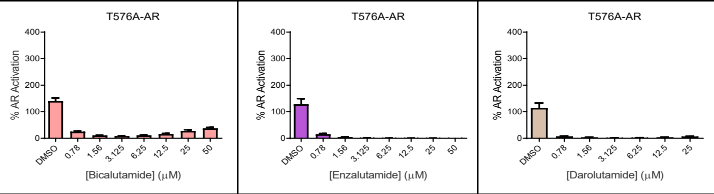		
K581R	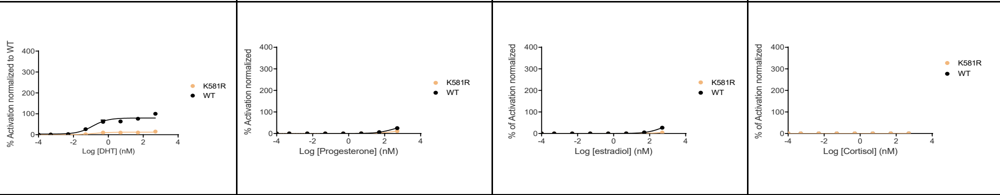				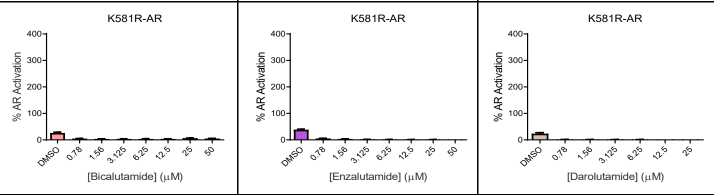		
A587V	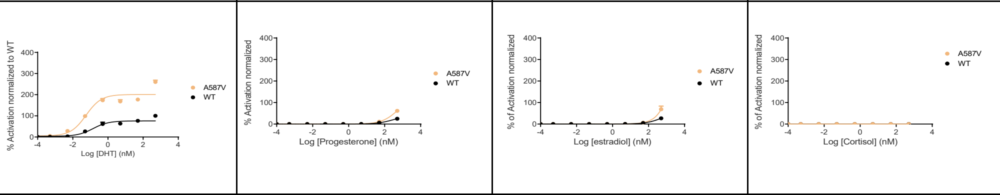				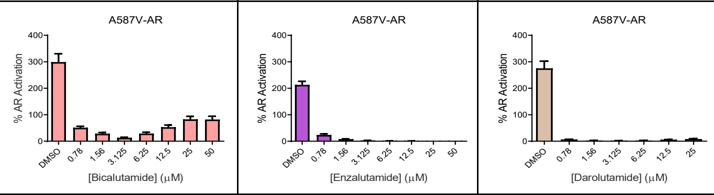		
A588S	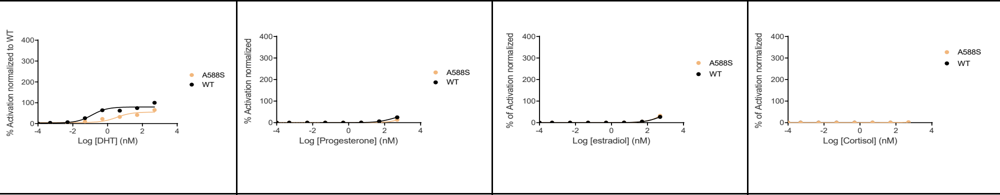				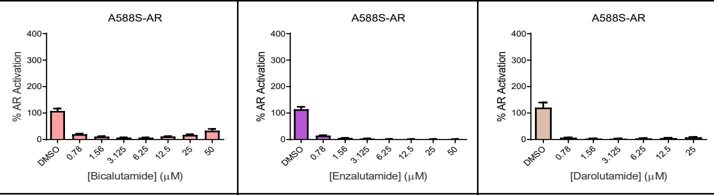		
L595M	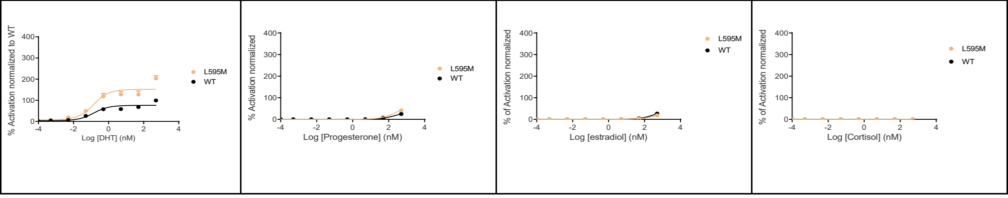				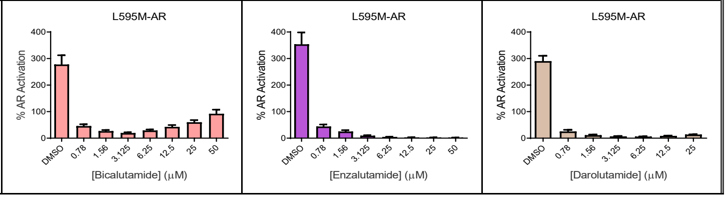		

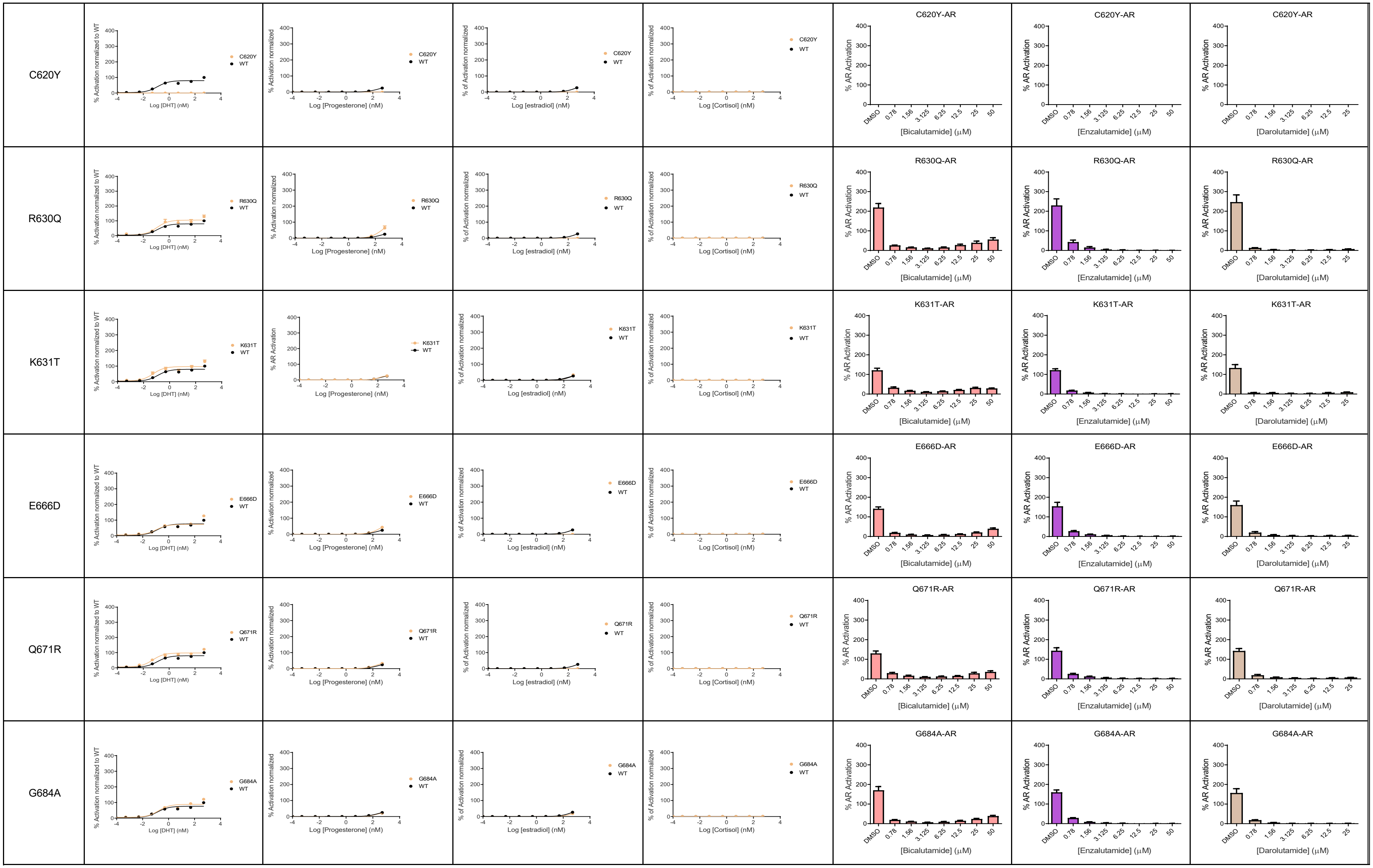
C620Y
R630Q
K631T
E666D
Q671R
G684A
% Activation normalized to WT
% Activation normalized
% of Activation normalized
% AR Activation
Log [DHT] (nM)
Log [Progesterone] (nM)
Log [estradiol] (nM)
Log [Cortisol] (nM)
WT
C620Y-AR
R630Q-AR
K631T-AR
E666D-AR
Q671R-AR
G684A-AR
% AR Activation
DMSO
0.78
1.56
3.125
6.25
12.5
25
50
[Bicalutamide] (μM)
[Enzalutamide] (μM)
[Darolutamide] (μM)

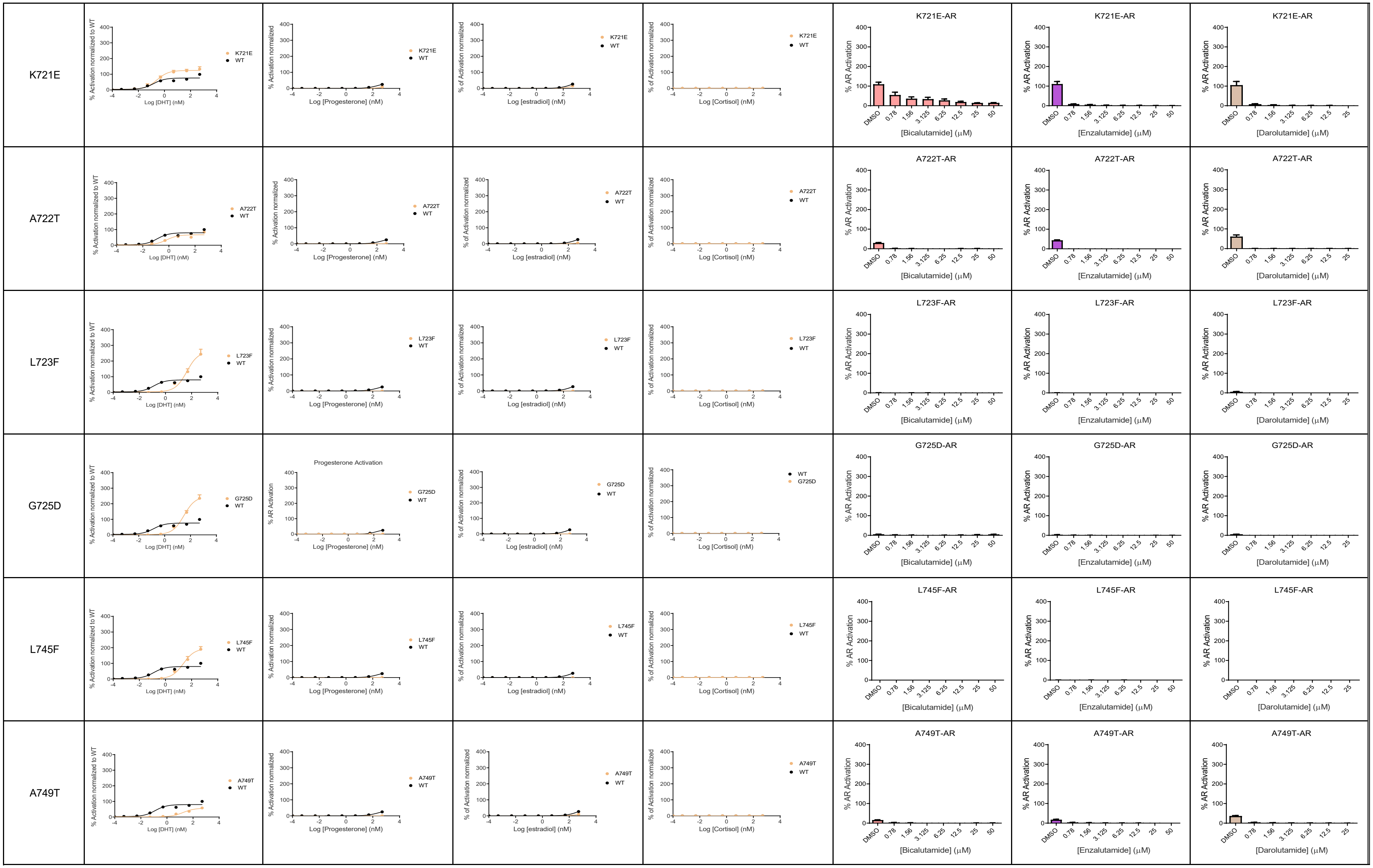
K721E
A722T
L723F
G725D
L745F
A749T
% Activation normalized to WT
Log [DHT] (nM)
% Activation normalized
% of Activation normalized
Log [Progesterone] (nM)
Log [estradiol] (nM)
Log [Cortisol] (nM)
Progesterone Activation
% AR Activation
WT
K721E-AR
A722T-AR
L723F-AR
G725D-AR
L745F-AR
A749T-AR
DMSO
0.78
1.56
3.125
6.25
12.5
25
50
[Bicalutamide] (μM)
[Enzalutamide] (μM)
[Darolutamide] (μM)

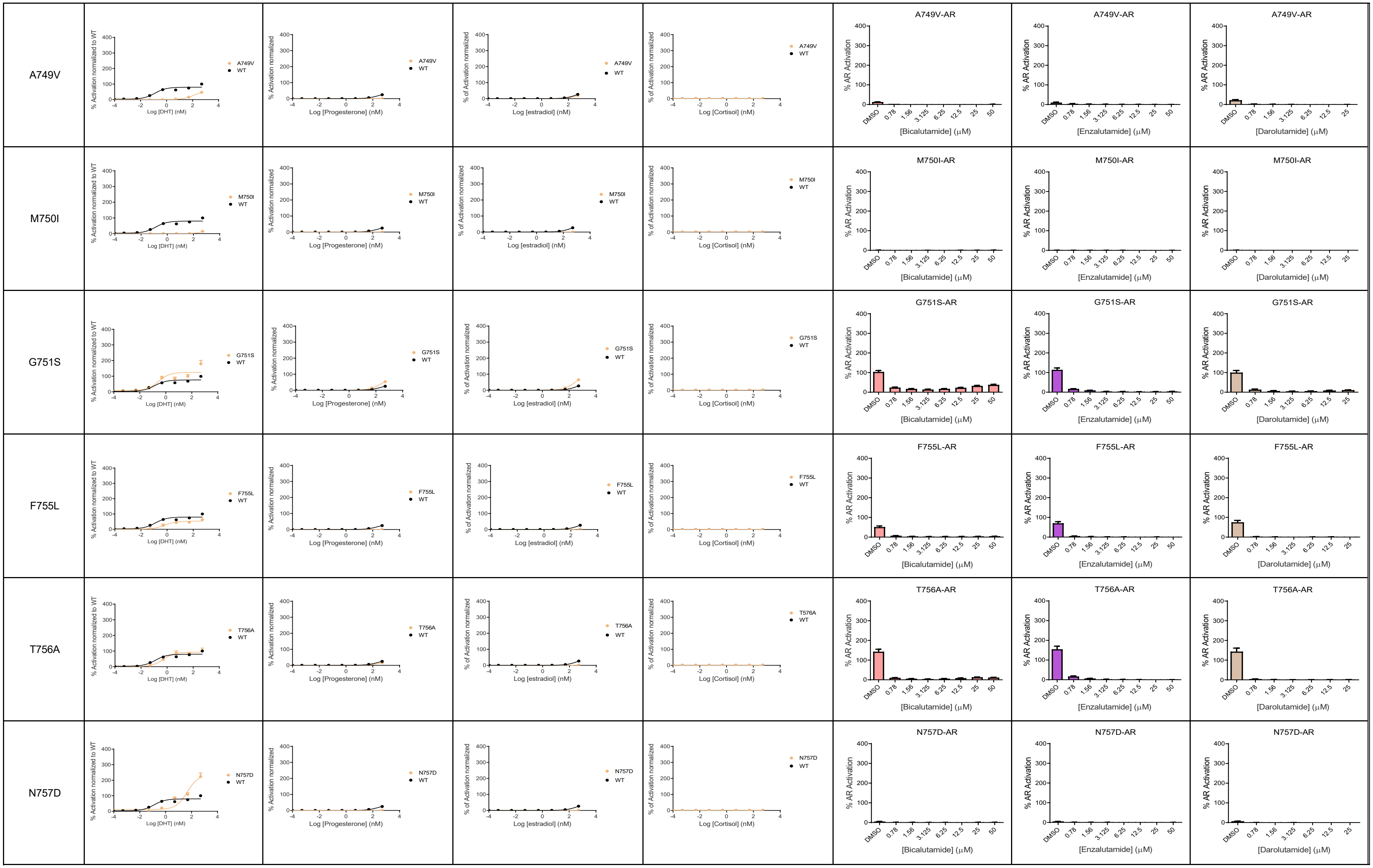
A749V
M750I
G751S
F755L
T756A
N757D
% Activation normalized to WT
Log [DHT] (nM)
% Activation normalized
Log [Progesterone] (nM)
% of Activation normalized
Log [estradiol] (nM)
Log [Cortisol] (nM)
WT
T576A
A749V-AR
M750I-AR
G751S-AR
F755L-AR
T756A-AR
N757D-AR
% AR Activation
[Bicalutamide] (μM)
[Enzalutamide] (μM)
[Darolutamide] (μM)
DMSO
0.78
1.56
3.125
6.25
12.5
25
50

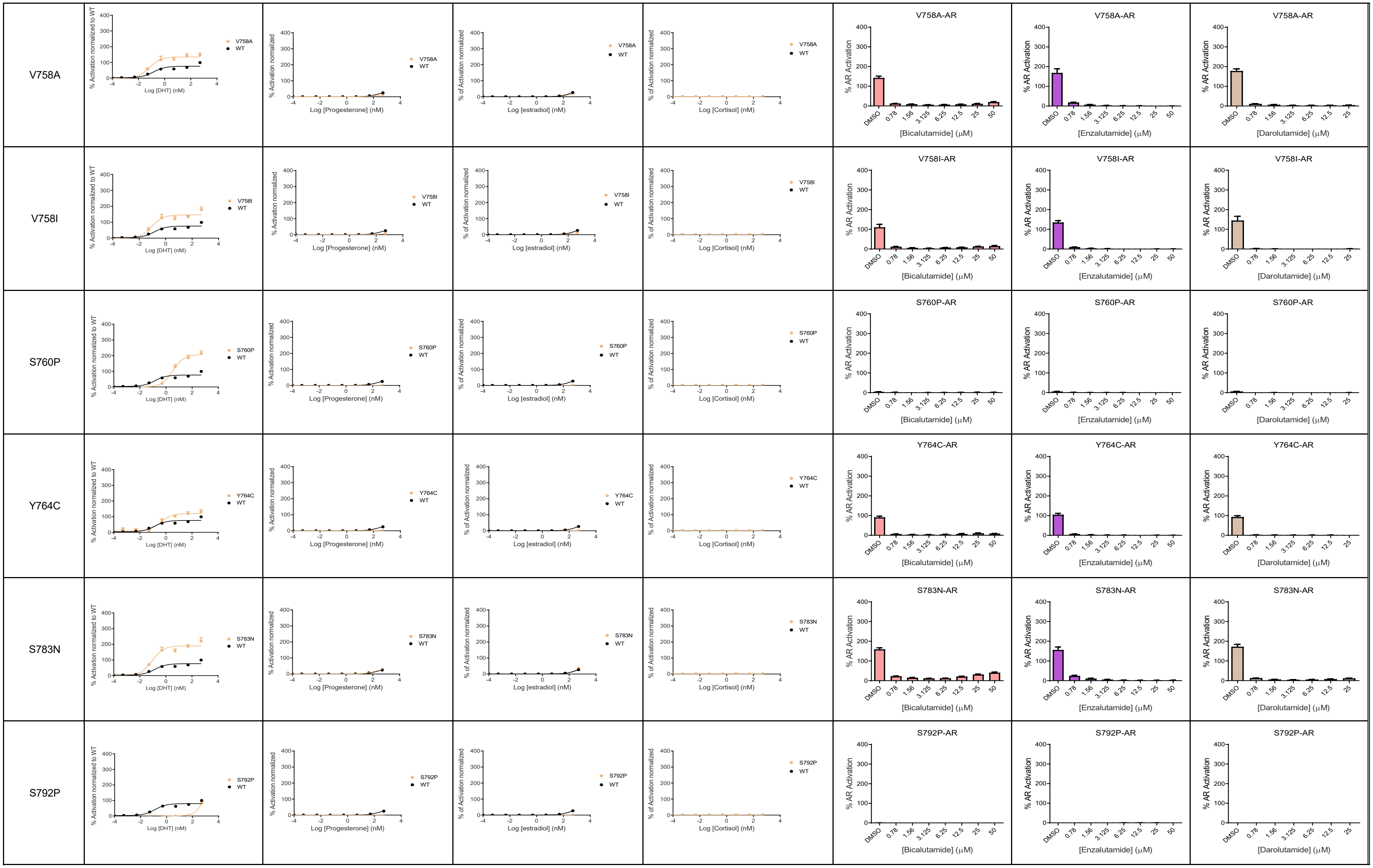
V758A
% Activation normalized to WT
Log [DHT] (nM)
V758A
WT
% Activation normalized
Log [Progesterone] (nM)
% of Activation normalized
Log [estradiol] (nM)
Log [Cortisol] (nM)
V758A-AR
% AR Activation
DMSO
0.78
1.56
3.125
6.25
12.5
25
50
[Bicalutamide] (μM)
[Enzalutamide] (μM)
[Darolutamide] (μM)
V758I
V758I-AR
S760P
S760P-AR
Y764C
Y764C-AR
S783N
S783N-AR
S792P
S792P-AR

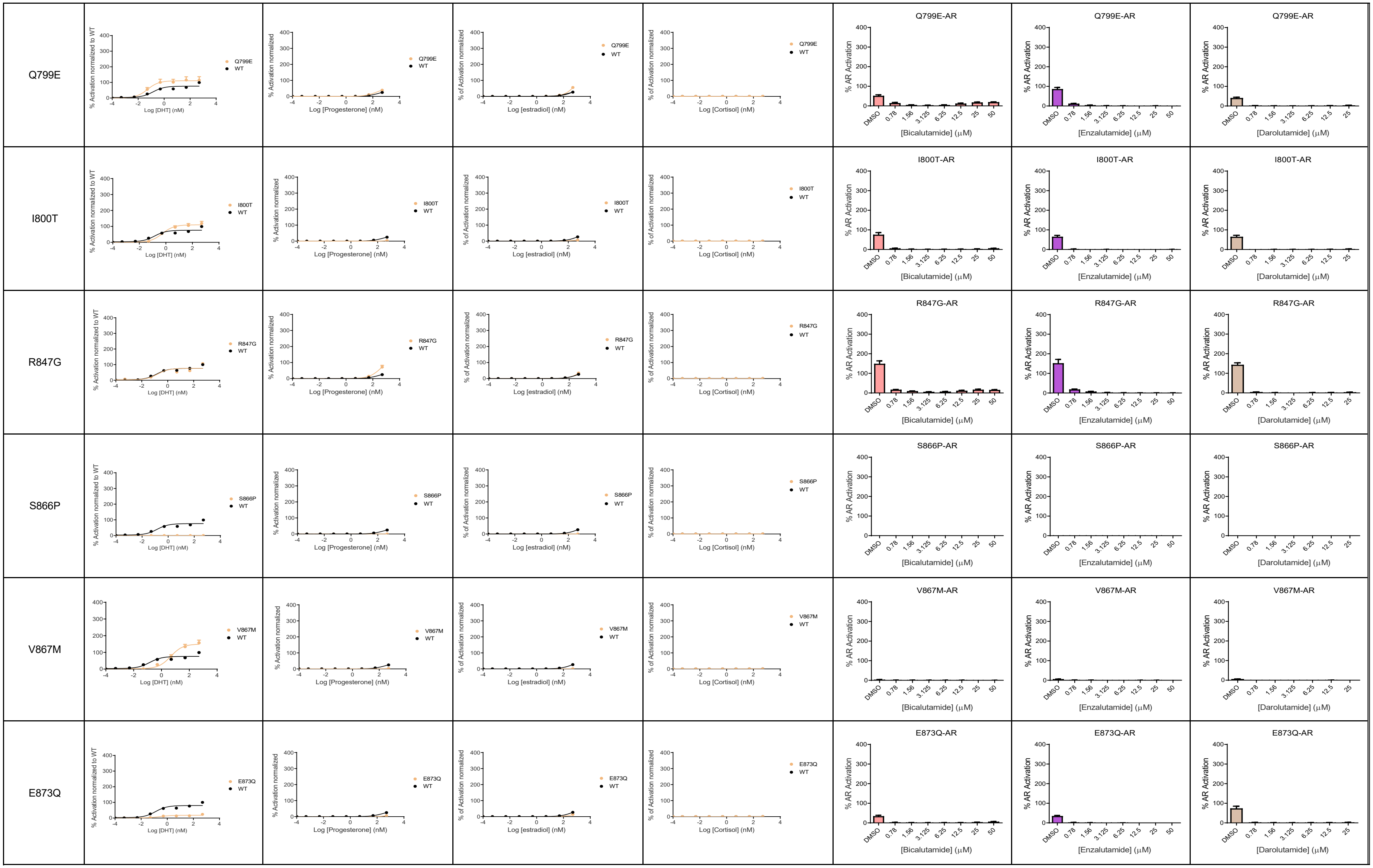
Q799E
I800T
R847G
S866P
V867M
E873Q
% Activation normalized to WT
% Activation normalized
% of Activation normalized
Log [DHT] (nM)
Log [Progesterone] (nM)
Log [estradiol] (nM)
Log [Cortisol] (nM)
WT
Q799E-AR
I800T-AR
R847G-AR
S866P-AR
V867M-AR
E873Q-AR
% AR Activation
DMSO
0.78
1.56
3.125
6.25
12.5
25
50
[Bicalutamide] (μM)
[Enzalutamide] (μM)
[Darolutamide] (μM)

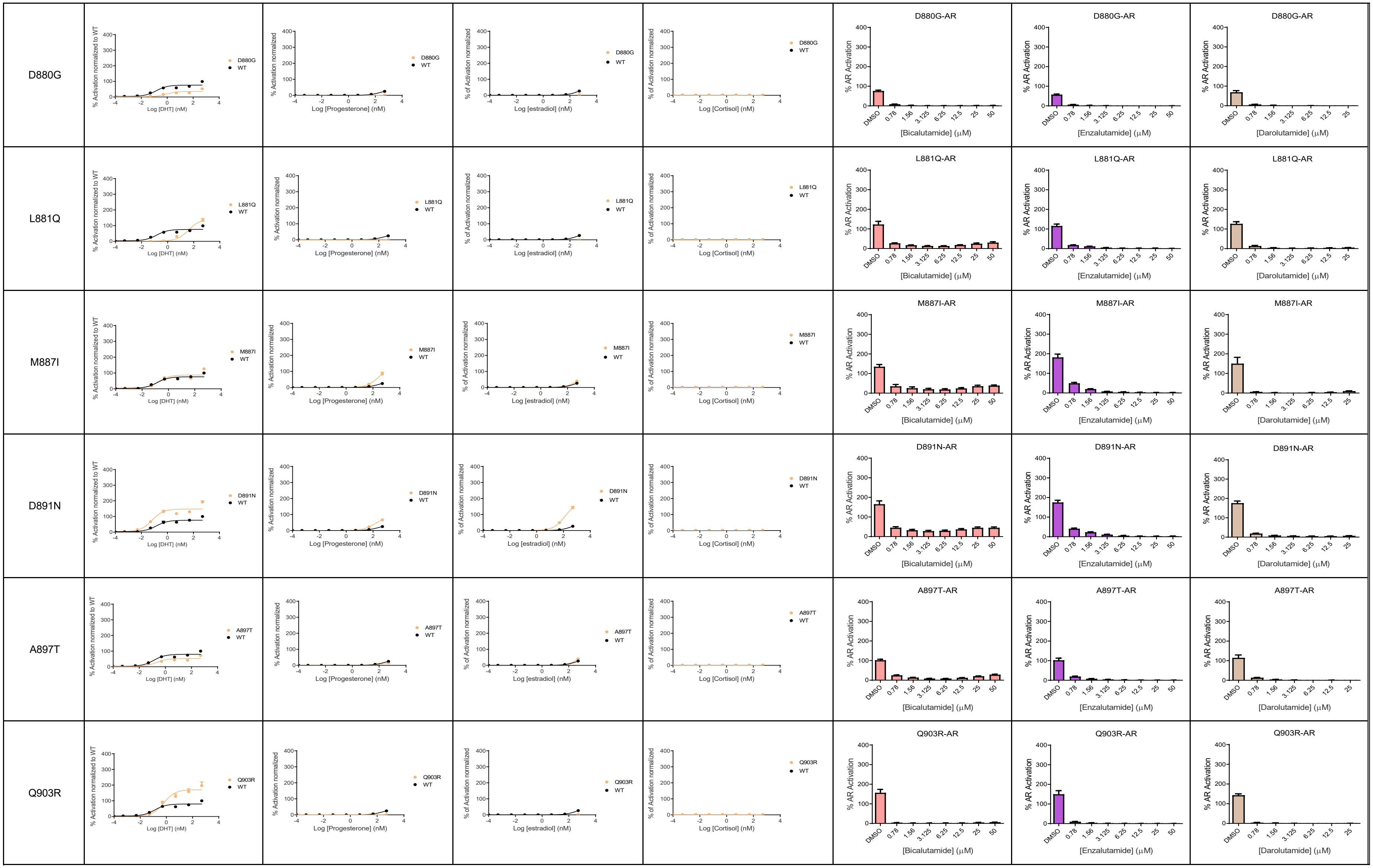

D880G
L881Q
M887I
D891N
A897T
Q903R
% Activation normalized to WT
Log [DHT] (nM)
% Activation normalized
Log [Progesterone] (nM)
% of Activation normalized
Log [estradiol] (nM)
Log [Cortisol] (nM)
WT
D880G-AR
L881Q-AR
M887I-AR
D891N-AR
A897T-AR
Q903R-AR
% AR Activation
DMSO
0.78
1.56
3.125
6.25
12.5
25
50
[Bicalutamide] (μM)
[Enzalutamide] (μM)
[Darolutamide] (μM)

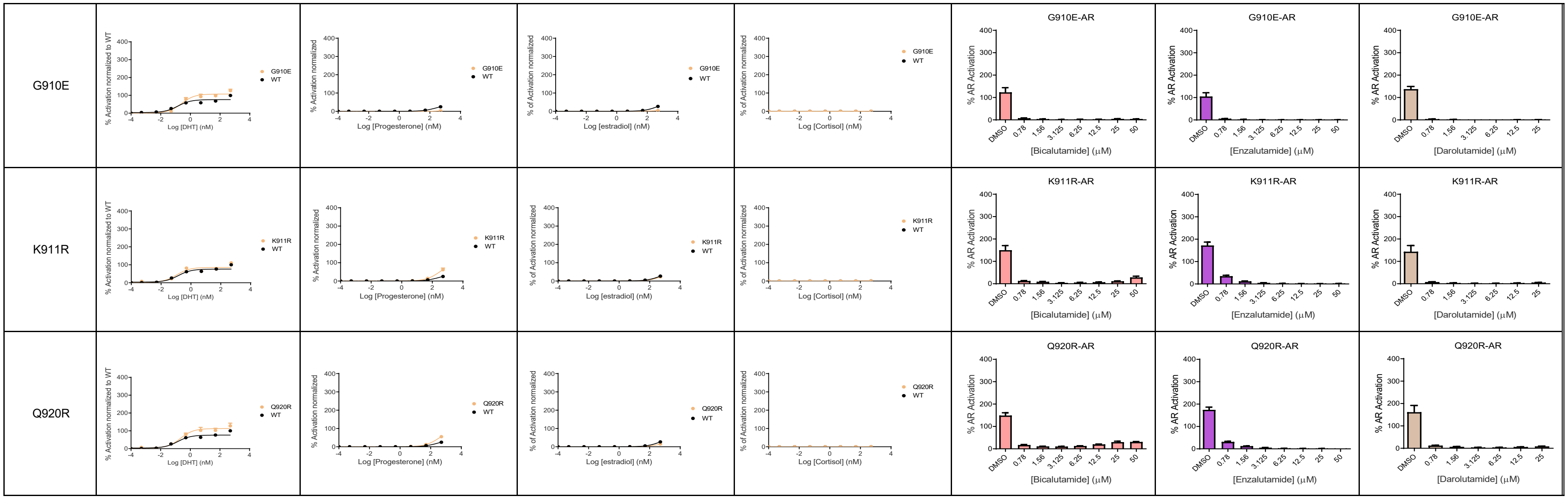

G910E
K911R
Q920R
% Activation normalized to WT
% Activation normalized
% of Activation normalized
Log [DHT] (nM)
Log [Progesterone] (nM)
Log [estradiol] (nM)
Log [Cortisol] (nM)
WT
G910E-AR
K911R-AR
Q920R-AR
% AR Activation
DMSO
[Bicalutamide] (μM)
[Enzalutamide] (μM)
[Darolutamide] (μM)
