# Supplementary figures and images for: Evaluation of Darolutamide (ODM201) Efficiency on Androgen Receptor Mutants Reported to Date in Prostate Cancer Patients
Source: Cancers (Basel). 2021 Jun 11;13(12):2939. doi: 10.3390/cancers13122939 (PMC8230763; doi:10.3390/cancers13122939)

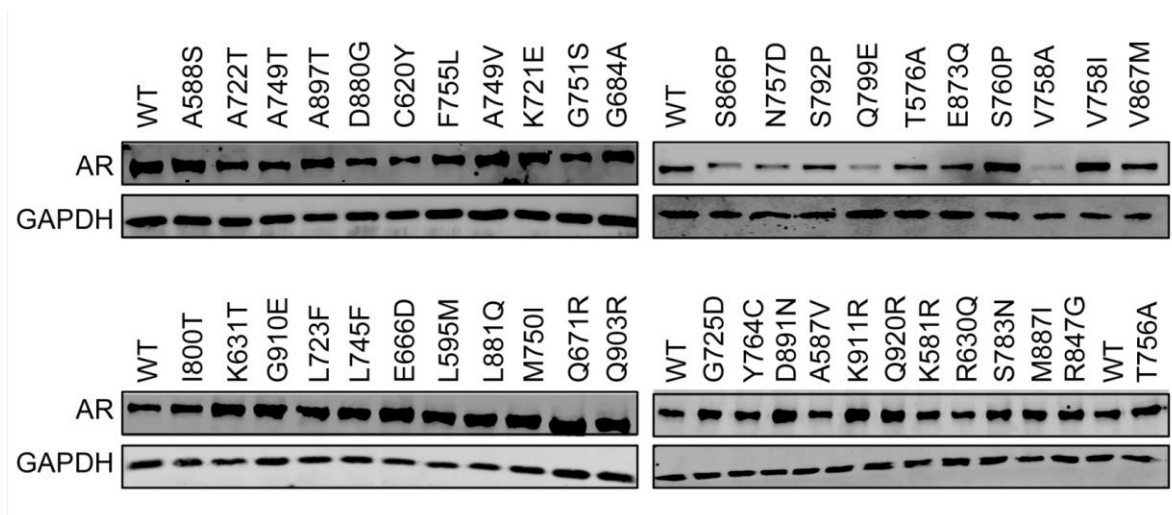

**Figure S1.** Western blot showing expression level of the PCa-associated AR mutants.

Supplement: Supplementary file 1 [file cancers-13-02939-s001.zip › cancers-1251001 Figure S1.pdf]
